# Design and development of synthetic microbial platform cells for bioenergy

**DOI:** 10.3389/fmicb.2013.00092

**Published:** 2013-04-19

**Authors:** Sang Jun Lee, Sang-Jae Lee, Dong-Woo Lee

**Affiliations:** ^1^Systems and Synthetic Biology Research Center, Korea Research Institute of Bioscience and BiotechnologyDaejeon, South Korea; ^2^School of Applied Biosciences, Kyungpook National UniversityDaegu, South Korea

**Keywords:** microbial platform cells, bioenergy, synthetic biology, genome reduction, metabolic engineering

## Abstract

The finite reservation of fossil fuels accelerates the necessity of development of renewable energy sources. Recent advances in synthetic biology encompassing systems biology and metabolic engineering enable us to engineer and/or create tailor made microorganisms to produce alternative biofuels for the future bio-era. For the efficient transformation of biomass to bioenergy, microbial cells need to be designed and engineered to maximize the performance of cellular metabolisms for the production of biofuels during energy flow. Toward this end, two different conceptual approaches have been applied for the development of platform cell factories: forward minimization and reverse engineering. From the context of naturally minimized genomes,non-essential energy-consuming pathways and/or related gene clusters could be progressively deleted to optimize cellular energy status for bioenergy production. Alternatively, incorporation of non-indigenous parts and/or modules including biomass-degrading enzymes, carbon uptake transporters, photosynthesis, CO_2_ fixation, and etc. into chassis microorganisms allows the platform cells to gain novel metabolic functions for bioenergy. This review focuses on the current progress in synthetic biology-aided pathway engineering in microbial cells and discusses its impact on the production of sustainable bioenergy.

## INTRODUCTION

Biological processes using microorganisms have a wide range of superior advantages (e.g., renewability, sustainability, and carbon neutralization) over conventional chemical processes for the production of biofuels. However, besides from compatibility with existing fuel infrastructure, production yields of the advanced biofuels are nevertheless not yet great enough to compete with and replace fossil fuels. The key issues to overcome in biological systems are the cost of substrates and biofuel toxicity/inhibition of fermenting microorganisms, which are directly related to biofuel productivity, titer, and yield (Ezeji et al., 2007; Fischer et al., 2008). To overcome these impediments, development of a robust and high-yielding microbe is required. Recently, a variety of engineered microorganisms, by metabolic engineering integrated with genome engineering and synthetic biology, appear to be quite promising with improved yields of biofuel production (e.g., ethanol, biodiesel, butanol, terpenoids, syngas, and H_2_; Bokinsky et al., 2011; Srirangan et al., 2011; Zhang et al., 2011; Lan and Liao, 2012; Li et al., 2012; Westfall et al., 2012).

Synthetic biology provides us with innovative approaches to a wide range of applications (Purnick and Weiss, 2009): sustainable bioenergy production, bioremediation, biorefinery, and biopharmaceuticals. Based on a wealth of genome sequences, systems biology and metabolic engineering integrated with advanced genetic tools have enabled us to make engineered microbes as a blueprint for the near future (Gibson et al., 2008a, 2009; Benders et al., 2010; Jewett and Forster, 2010). Indeed, not only artificial microorganisms based on parasites (Fraser et al., 1995) and small size-genome microorganisms (Gibson et al., 2008b, 2010), but also engineered microorganisms were successfully generated for the production of biofuels, fine chemicals, pharmaceuticals, and biosensors (Martin et al., 2003; Keasling, 2010; Huo et al., 2011; Zhang and Keasling, 2011; Zhang et al., 2011). Thus, construction of efficient biofuel-producing microbial cell factories is now conceivable by design-based engineering of biological systems (Forster and Church, 2006, 2007; Carothers et al., 2009; Nitschke, 2009; Holtz and Keasling, 2010; Nielsen and Keasling, 2011; Tolonen et al., 2011).

To design and engineer microorganisms for the high-yields of biofuel production, we need to better understand how microbial cells can coordinate their metabolic pathways under different environmental conditions, underlying essential and non-essential genes for bacterial life and metabolic networks. This information will help us to modulate the efficiency of production pathway and to optimize the energy balances between bioproduction and biosynthesis in cell factories. To date, there are increasing examples of engineering metabolic pathways tightly linked to the cellular energy balance that is one of the key determining factors of cell factories in the yield and productivity of biofuels (Johnson and Schmidt-Dannert, 2008; Trinh et al., 2008; Portnoy et al., 2010; Lan and Liao, 2011, 2012). Recently, molecular engineering using protein or RNA scaffolds also could be applied for pathway engineering in synthetic cell factories (Conrado et al., 2008; Delebecque et al., 2011; Medema et al., 2011). For example, organizing mevalonate pathway enzymes on scaffolds have been developed for efficient production of isoprenoids (Dueber et al., 2009). In this review, we will focus on the current strategies for designing and developing cell factories for the maximized production of sustainable bioenergy in the context of “forward engineering” and/or “reverse engineering” of efficiently energy-optimized cells.

## “TOP-DOWN” REDUCTION OF MICROBIAL GENOME BY FORWARD ENGINEERING

Recent advances in sequencing techniques have generated enormous amounts of microbial genome databases, which can be invaluable information for the physiology and metabolism of the sequenced microorganisms (Brochier-Armanet et al., 2011; Gonzalez and Knight, 2012). This information also provides insight into the diversity of microorganisms and the molecular basis of their adaptive evolutionary mechanisms in different environments (Forterre et al., 2000). Comparison of genomes can often reveal similar and/or distinctly different metabolic pathways in bacteria, archaea, and eukarya (Siebers and Schonheit, 2005; Brochier-Armanet et al., 2011). In particular, the genome sequences of diverse microorganisms including extremophiles showed genetic traits of adaptation through gene duplication and/or deletion (Riehle et al., 2001; Averhoff, 2009). It suggests that gain and loss of genes is one of the major adaptation mechanisms for their cellular viability under selection pressure in nature. Thus, the genome sequences of diverse microorganisms that have an ancestor in common have diverged in a variety of ways, indicating that specific genes of a microorganism that are not found in others and highly conserved genes among organisms can be feasibly categorized. Accordingly, this leads us to have a fundamental question of which genes are indispensable for cellular lives and are involved in their essential and distinct metabolisms in comparison with other microbes. Moreover, these fundamental informations can provide indirect but very essential knowledge to overcome the endogenous regulation of biofuel-producing pathways to achieve high yields in using native hosts to convert feedstocks into biofuels.

### GENOME COMPARISON OF ARCHAEA

**FIGURE 1:**
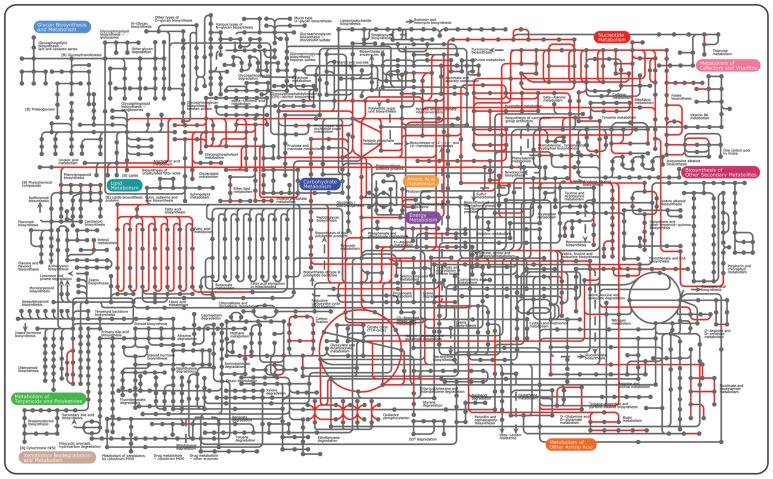
**Overview of the microbial pathways on the KEGG pathways using the iPath tool (Letunic et al., 2008**. To date, conserved pathways known as essential are shown in red. Hypothetical proteins found as essential are excluded.

Minimal cells comprise only the genes and biomolecular machineries required for basic life. During the past decade, over 100 genomes of archaea that form the third domain of life (Brochier-Armanet et al., 2011) have been sequenced. In fact, their genome data provide insights into the evolution of key central metabolisms, which are directly correlated with the minimal functionality for cell viability and adaptation under extreme environments. Intriguingly, the euryarchaea *Picrophilus torridus*, which thrives optimally at 60°C and pH 0.7, has 1.55 Mb genome (1535 ORFs), the smallest genome among non-parasitic aerobic microorganisms (Futterer et al., 2004). In particular, an exceptionally high ratio (5.6:1) of secondary over ATP-consuming primary transport systems represents a highly relevant strategy for the adaptation of this organism to its extremely acidic environment. Although *Picrophilus torridus* has several distinct gene traits in energy metabolism at low pH, not only all genes required for the Embden–Meyerhof–Parnas (EMP) pathway but also a complete set of genes for the oxidative tricarboxylic acid (TCA) cycle are present. Currently, there is a variety of genome data available for other archaea and bacteria, such as *Thermoplasma acidophilum* (1.56 Mb; Ruepp et al., 2000), *Archaeoglobus profundus* (1.56 Mb; von Jan et al., 2010), *Prochlorococcus marinus* (1.75 Mb; Dewall and Cheng, 2011),**and *Methanocaldococcus jannaschii* (1.74 Mb; Bult et al., 1996), of which genome size is less than 2 Mb. Thus, these microorganisms seem to be “closer” to the minimal genome for life, which will also provide the fundamental basis of minimal gene sets for the construction of a genome-minimized platform cell (**Figure 1**).If this is the case, can we design and construct an engineered platform cell to perform our wanted tasks such as biofuel production? If so, then can we selectively choose which genes are friends or foes for the high productivity between cell mass and biofuels? Prior to considering this issue, we need to categorize essential sets of genes for cellular viability even with imperfect accuracy.

### GENOME OF PHOTO-AUTOTROPHIC AND -HETEROTROPHIC CYANOBACTERIA

Genes involved in informational processes (not only DNA replication, but also transcription and translation represented by rRNA, tRNA, or structural RNA genes) plus protein folding and processing with strain-dependent metabolisms, are essential. In addition, a complex array of functional systems, including those for membrane transport, energy conversion, the synthesis of vitamins, and nucleic acid precursors, is indispensable for maintaining cellular integrity. Notable examples are the marine cyanobacteria *Prochlorococcus*, which is the smallest known oxygen-evolving photoautotroph (Chisholm et al., 1988). Although the high light-adapted ecotype MED4 strain has a genome of 1.66 Mb that codes for 1717 proteins, its low light-adapted counterpart contains a larger genome of 2.4 Mb (2275 genes; Rocap et al., 2003). Remarkably, the comparative analysis with their genomes revealed that only 1350 genes are common, whereas the remaining genetic loci are quite different, implying that the variable genes appear to be a consequence of a selective process favoring the bacterial adaptation to their environments (Dufresne et al., 2003, 2005; Rocap et al., 2003). Indeed, the photoheterotrophic marine bacterium *Pelagibacter ubique* has the smallest genome (1.3 Mb) of any cell known for a free-living microorganism (Giovannoni et al., 2005). Its genome coding for 1354 ORF shows the nearly complete absence of non-functional or redundant DNA, with very short intergenic regions, and the lack of pseudogenes and phage genes, reflecting an adaptive strategy that resembles the highly successful marine unicellular cyanobacteria in its simple metabolism and small genome size.

### NON-ESSENTIAL GENES

Synthesizing minimal and minimized cells will improve understanding of core biology, accelerate development of biotechnology strains of bacteria, and enable evolutionary optimization of natural and unnatural biopolymers (Jewett and Forster, 2010). Genome reduction is of particular importance to identify non-essential genes for understanding of not only how many genes are essential for cellular viability, but also which genes are necessary for cellular beneficial properties. Reduction and engineering of microbial genome is the fundamental basis of design and development of synthetic minimal platform cells for estimation of the minimal gene set required to sustain growth of microorganisms (Fleischmann et al., 1995; Fraser et al., 1995; Mushegian and Koonin, 1996). By use of comparative genomics, non-essential genes have been sought to reconstruct ancestral life forms (Mushegian and Koonin, 1996; Koonin, 2003) to define, by transposon-mediated disruption study, with *Mycoplasma genitalium *(Glass et al., 2006), and to validate and compare the minimal gene sets in *Bacillus subtilis *(Kobayashi et al., 2003; Ara et al., 2007), *E. coli *(Gerdes et al., 2003; Joyce et al., 2006), *Aquifex aeolicus *(Deckert et al., 1998), *Streptococcus sanguinis *(Xu et al., 2011), and Yeast (Kellis et al., 2003, 2004). Although symbiotic archeal organisms apparently have a much smaller genome (Huber et al., 2002; McCutcheon and Moran, 2012), these are out of scope with respect to the minimal gene-set that is necessary and sufficient to support cellular life. Notably, toxin–antitoxin (TA) loci functioning as stress-response elements are not generally essential to basic life (Pandey and Gerdes, 2005). Yet, combined mutations in two or more genes lead to cell death, whereas a single mutation in only one of those genes does not (Tucker and Fields, 2003). Thus, synthetic lethality should be considered when non-essential genes are deleted for genome minimization (Stieber et al., 2008).

### MINIMAL GENOME FOR SYNTHETIC CELLS

Intriguingly, Posfai et al. (2006) successfully reduced the *E. coli* K-12 genome (up to 15%) to generate multiple-deletion series of strains without physiological compromise. In addition, genome reduction would provide unexpected benefits, such as high electroporation efficiency and accurate propagation of recombinant plasmids. Several laboratory evolution studies have also been carried out to characterize the genetic traits of adaptation to environmental stresses (e.g., thermal adaptation, salt stress tolerance, utilization of unusual substrates, and susceptibility to antibiotics, etc.; Herring et al., 2006; Dhar et al., 2011; Tran et al., 2011). Remarkably, reduction of the metabolic pathways by selective sorting and deletion of aerobic/anaerobic reactions based on the biomass and biofuel productions enabled cells to have theoretically maximal yields of ethanol even with minimized metabolic functionality under anaerobic conditions (Trinh et al., 2006, 2008). To convert the biomass-derived hexoses and pentoses to ethanol at high yields and productivities, an efficient and robust microorganism has been designed and developed by the removal of seemingly unnecessary pathways for the purpose (Trinh et al., 2006). To construct a minimal *E. coli* cell that is dedicated to producing ethanol, the functional space of the central metabolic network was reduced with eight gene knockout mutations (e.g., *poxB*, *pta* etc.) from over 15,000 pathway possibilities to 6 pathway options that support cell function (Trinh et al., 2008). The remaining pathways consist of four pathways with non-growth-associated conversion of pentoses and hexoses into ethanol at theoretical yields and two pathways with tight coupling of anaerobic cell growth with ethanol formation at high yields. Remarkably, catabolite repression was completely absent during anaerobic growth by the deletion of acetate-producing pathways, resulting in the simultaneous utilization of pentoses and hexoses for ethanol production in the most efficient way. Thus, this study demonstrated that the ethanol yields of engineered strains with minimized metabolic functionality closely matched the theoretical predictions, implying that reduction of non-essential genes could be quite beneficial with respect to economical production yields in synthetic microorganisms.

## “BOTTOM-UP” CREATION OF MICROBIAL CELL FACTORY BY REVERSE ENGINEERING

To avoid and overcome our coming issues with global warming and energy crises, increasing efforts to replace fossil fuels with renewable biofuels are still actively being undertaken in biotechnology fields, resulting in several renewable alternatives such as bioalcohol and plant oil-derived biodiesel from biomass feedstock (Atsumi et al., 2008; Nielsen et al., 2009; Lan and Liao, 2011; Wargacki et al., 2012). Recently, First generation biofuels such as ethanol and biodiesel have provided an avenue to sustainable biofuels in the near future, but they also appear to have their own limitations, such as a lower efficiency of combustion (Gray et al., 2006; Chisti, 2008). Thus, scientists are seeking to develop more sustainable and economically feasible second generation microbial biofuels (i.e., butanol, hydrocarbon, alkanes, H_2_), which have great potential to convert renewable sources into energy rich, fuel-like molecules or fuel precursors (Schirmer et al., 2010; Steen et al., 2010; Shen et al., 2011). Many efforts in the fields of metabolic engineering, systems biology, synthetic biology, and genome engineering for biofuel production enable us to modulate indigenous metabolic fluxes, or to insert novel pathways by employing heterologous ones into host microorganisms for (i) increased productivity/titer****of biofuels and (ii) energy-efficiency of metabolic pathways. In fact, the former effect is highly dependent on the substrate utilization and tolerance of host cells for product inhibition (Papoutsakis, 2008; Geddes et al., 2011; Peralta-Yahya et al., 2012). On the other hand, the latter seems to be much more important for significantly improving the overall productivity, because it is tightly coordinated with cellular energy transduction through redox homeostasis in cells under specific conditions (Martin et al., 2003; Trinh et al., 2008; Lan and Liao, 2012). Thus, design of microbial platform cells for high yields of biofuel production requires understanding how energy transduction systems including respiratory chains are partitioned and matched stoichiometrically with central metabolisms. Moreover, when foreign energy transduction pathways are employed, they should be compatible with indigenous central and energy metabolisms in host cells. Indeed, this is major challenges in improving the kinetics of metabolic enzymes and generating metabolic driving forces to maximize metabolic flux. In light of this, less genetically tractable hosts that have high biofuel tolerance or the ability to use non-sugar substrates are potentially applicable alternatives. This requires not only whole genome sequences of platform cells but also genome-wide systems analysis. Recent advent of next-generation DNA sequencing expands the width of genomic diversity and allows the exploitation of a variety of novel metabolic enzymes/pathways.

### GENOME ENGINEERING TOOLS

To date, a wealth of gene disruption and shuttle vector systems has been developed for *E. coli* as a model system. Simple inactivation of chromosomal genes by the PCR-mediated gene replacement (Datsenko and Wanner, 2000) has greatly facilitated the generation of specific mutants in the functional analysis of the microbial genome. It has been noted that disseminated throughout the genome are mobile DNA elements, which mediate recombination events, such as transposition and horizontal gene transfer, including insertion sequence (IS) elements, transposases, defective phages, integrases, and site-specific recombinases (Frost et al., 2005). To stabilize the genome and streamline metabolism, these elements must be deleted and unwanted functions removed. These unwanted functions include those specific for human hosts or particular environments. Indeed, a Tn5-targeted Cre/IoxP excision system (Yu et al., 2002) and a high-throughput method for the systematic mutagenesis of the genome by Tn5 transposon (Kang et al., 2004) enabled us to create individual *E. coli* deletion and insertion mutants without loss of normal growths. In particular, a multiplex automated genome engineering (MAGE) technique as a powerful high-throughput genome engineering tool has been developed (Wang et al., 2009a). MAGE simultaneously targets many locations on the chromosome for modification in a single cell or across a population of cells using allelic replacement to produce combinatorial genomic diversity through an oligonucleotide-directed recombineering technique. In addition, precise manipulation of chromosomes *in vivo* enables genome-wide codon replacement as well (Isaacs et al., 2011). Further, as described above genome reductions may improve metabolic efficiency and decrease the redundancy among microbial genes and regulatory circuits (Posfai et al., 2006). Therefore, a rational design allows us to attempt to delete genes more extensively while avoiding loss of robustness, which can allow a chassis cell to be incorporated with biofuel-producing synthetic and/or engineered pathways.

### OPTIMIZATION OF CELLULAR ENERGY METABOLISM

The attractive strategy described above immediately tempts us to ask questions like “Can we selectively delete the alternative energy transduction pathways as an engineering target?” and “Are minimal sets of genes beneficial in terms of energy efficiency?” This might be true simply because extreme environments already show a variety of extremophiles that can grow optimally with relatively small genome sizes (less than 2 Mb), as discussed above. In fact, their energy metabolisms appear to be designed and adapted to survive under their own specific environments in the most minimal but efficient way. This rationale seems to be supported by several challenging experiments about the effect of the modulation of respiratory chains on fermentation products (Portnoy et al., 2008, 2010). In these studies, a series of aerobic respiratory chains in *E. coli* were progressively knocked out and aerobically adapted to generate an evolved mutant deficient in three terminal oxidases. Initially, this mutant could not grow on M9 minimal medium containing D-glucose. However, 60-day adaptive evolution on the same medium created *E. coli* mutants that exhibited the ability to undergo mixed-acid fermentation during aerobic growth and to produce lactate as a fermentation product from D-glucose. Moreover, the removal of three terminal cytochrome oxidase genes (*cydAB*, *cyoABCD*, and *cbdAB*) and a quinol monooxygenase gene (*ygiN*) yielded an *E. coli* mutant that exhibits anaerobic physiology even in the presence of oxygen, through the activation of the anoxic redox control transcriptional regulator ArcA (Portnoy et al., 2010). The knockout strain exhibited nearly identical physiological behaviors and produced D-lactate as the sole by-product under oxic and anoxic conditions, suggesting that the mutations resulted in significant metabolic and regulatory perturbations.

Genome-scale transcriptome analysis, ^13^C tracing experiments, and physiological characterization demonstrated that the deletions resulted in the activation of anaerobic respiration under oxic conditions and a consequential shift in the content of the quinone pool (Portnoy et al., 2010). This result suggests that composition of quinone pool may be tightly coordinated with the activation of ArcB/ArcA regulatory system, concomitantly linked to a major shift in the metabolic flux distribution through the central metabolism in cells (Alexeeva et al., 2000). Therefore, respiratory control and/or modulation could be an efficient engineering strategy for changing the central metabolic flux of cells for the high yields of biofuel production (Fischer et al., 2008; Portnoy et al., 2010). This strategy can be validated by a systems-level characterization of fermentative profiles, using single gene knockouts in *E. coli*, which are related to redox reactions (Lee and Lee, unpublished data). The data demonstrated that single gene deletion mutations in *guaB*, *pyrD*, and *serA *increased D-lactic acid production. Combined knockouts of *guaB*, *pyrD*, *serA*, *fnr*, *arcA*, or *arcB* further enhanced D-lactate production.

Very recently, the hyperthermophilic archaeon *Pyrococcus furiosus* with the small genome size of 1.91 Mb, which grows optimally at 100°C, can be engineered to produce important organic chemicals from CO_2_ by use of low potential reducing power from H_2_ (Keller et al., 2013). For this, five genes of the carbon fixation cycle of the archaeon *Metallosphaera sedula,* which grows autotrophically at 73°C were heterologously expressed in *Pyrococcus furiosus*. The engineered *Pyrococcus furiosus* strain is able to use H_2_ and incorporate CO_2_ into 3-hydroxypropionic acid, a chemical building block for the production of acrylic acid, acrylamide, and 1,3-propanediol. Remarkably, this is operated at temperatures that are suboptimal for its growth to minimize the metabolic burden of the engineered microorganisms during chemical production from H_2_ and CO_2_. Such a strategic operation support only minimal growth but maintain sufficient metabolic activity to sustain the production of 3-hydroxypropionate. The unique temperature-dependent approach (Basen et al., 2012) that confers on a microorganism the capacity to use CO_2_, a reaction that it does not occur naturally circumvents the overall low efficiency of photosynthesis and the production of sugar intermediates.

## BIOMASS-BASED BIOFUEL PRODUCTION BY ENGINEERED MICROORGANISMS

**FIGURE 2:**
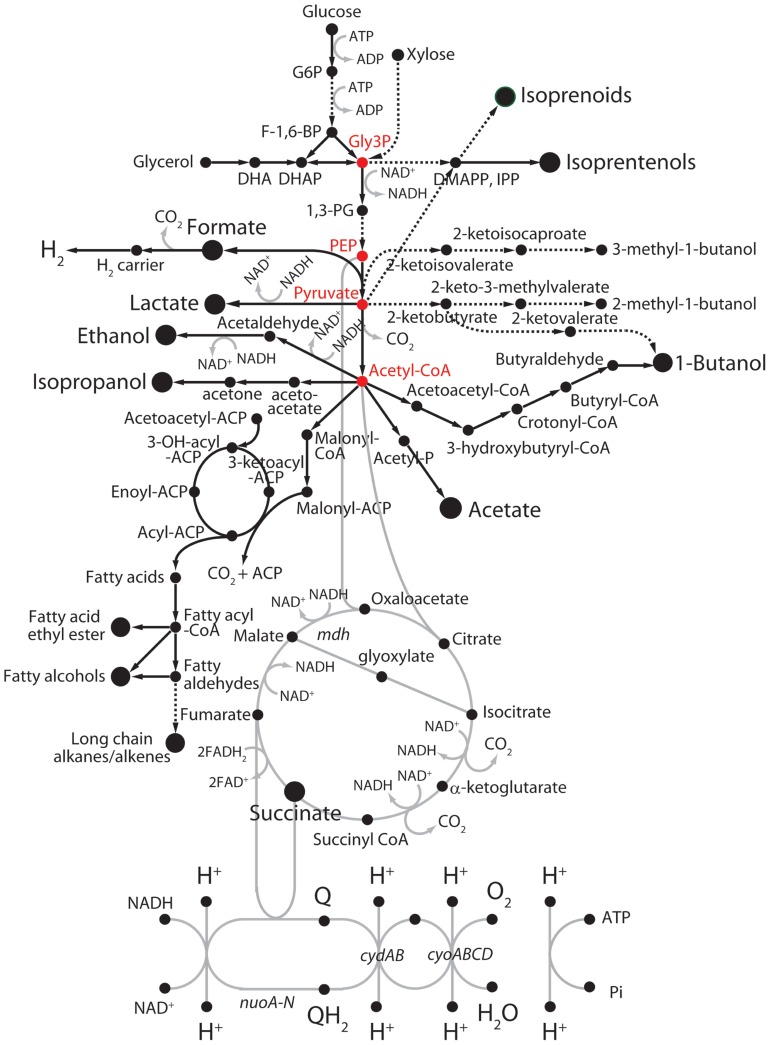
**Fermentative and non-fermentative pathways for the production of biofuels in *E. coli***. Dashed lines represent multiple reaction steps. Red circles represent metabolic intermediates.****ACP, acyl-carrier protein; DHAP, dihydroxyacetone phosphate; DMAPP, dimethylallyl pyrophosphate; F-1,6-BP, fructose-1,6-bisphosphate; G6P, glucose-6-phosphate; Gly3P, glyceraldehyde 3-phosphate; IPP, isopentenyl pyrophosphate; 1,3-PG, 1,3-diphosphoglycerate; PEP, phosphoenolpyruvate.

A wide variety of metabolic engineering and systems biology approaches, including synthetic biology with microorganisms, has been made for exploitation of diverse biomass resources (Walter et al., 2007; Rude and Schirmer, 2009; Oh et al., 2011; Zhang et al., 2011). Although these approaches are promising, there are still limitations in terms of the technical feasibility of cost-effective energy resources and the availability of rapid genetic tools and in-depth physiological knowledge for the effective manipulation of energy transduction systems. Accordingly, a variety of research works have been focused on the development of a cost-effective and energy-efficient engineered microorganisms as platform cells to produce biofuels****(Nakamura and Whited, 2003; Johnson and Schmidt-Dannert, 2008; Nielsen et al., 2009; Bokinsky et al., 2011; Yim et al., 2011). Implementation of heterologous pathways and/or metabolisms by the incorporation of single enzyme or a metabolic pathway module into host platform cells is the most frequently used strategy (Johnson et al., 2010; Hopkins et al., 2011; Shen et al., 2011; Lan and Liao, 2012; Westfall et al., 2012). With a macroscopic aspect, cellular metabolisms can be divided into two metabolic pathways: “feed” and “production” pathways (Fischer et al., 2008). Toward the high yields of titer and productivity in an engineered platform cell, tuning the redox balance between central energy and carbon metabolisms via metabolic intermediates is a key factor to improve biofuel productivity, while preventing from the reduction of bioenergetic waste reactions. As shown in **Figure 2**, microbial pathways for production of biofuels were categorized into four subgroups: non-fermentative alcohols, fatty acid-derived hydrocarbons, isoprenoid-derived hydrocarbons, and fermentative alcohols (Rude and Schirmer, 2009).

### CELLULOSIC BIOFUELS

In contrast to starches and simple sugars derived from sugar cane and corn, lignocellulosic crops are regarded as sustainable and renewable. However, they also have some technical hurdles due to recalcitrance of cellulosic biomass rich in lignin, resistance to enzymatic hydrolysis and the presence of five carbon sugars (Demain et al., 2005). In this respect, consolidated bioprocessing is a promising strategy to overcome biomass recalcitrance by using cellulolytic microorganisms. One of the most closely studied of the cellulolytic microbes, *Clostridium thermocellum* (Wiegel et al., 1985), is being used for the production of ethanol through the consolidated bioprocessing of plant biomass (Bayer et al., 2008; Lynd et al., 2008). Recently, several genome sequences of thermophilic, plant biomass-degrading members of this genus (Blumer-Schuette et al., 2011) indicate that significant differences in glycoside hydrolase inventories and numbers of carbohydrate transporters exist, which likely relates to variability observed in plant biomass degradation capacity (Dam et al., 2011). In addition, the proteomic analysis**of* C. phytofermentans*, which contains 161 carbohydrate-active enzymes, has been performed to identify hydrolases and metabolic enzymes to engineer microorganisms for improved cellulosic bioconversion (Tolonen et al., 2011). Intriguingly, it has been found that increase in tryptophan and nicotinamide synthesis was entailed with cellulosic fermentation for the production of ethanol, providing novel genetic targets for more efficient conversion of biomass to fuels and biomaterials (Tolonen et al., 2011). Alternatively, degradation of cellulosic biomass by extremely thermophilic bacteria *Caldicellulosiruptor* strains could have high potential for the production of biofuels (Blumer-Schuette et al., 2011).

Moreover, several attempts to tackle these practical issues to promote biomass waste solutions and biofuel production have been made through metabolic engineering and synthetic biology (Himmel et al., 2007; Vazana et al., 2012). To efficiently degrade crystalline cellulose, artificial enzymatic cellulosome complexes were designed and examined. There are an increasing number of research papers describing the production of designer cellulosomes either *in vitro, ex vivo, *or* in vivo* (Vazana et al., 2012). In designer cellulosomes, each enzyme is equipped with a dockerin module that interacts specifically with one of the cohesin modules of the chimeric scaffoldin. Artificial scaffoldins serve as docking backbones and contain a cellulose-specific carbohydrate-binding module that directs the enzymatic complex to the cellulosic substrate, as well as one or more cohesin modules from different natural cellulosomal species, each exhibiting a different specificity that allows the specific incorporation of the desired matching dockerin-bearing enzymes (Demain et al., 2005).

Another interesting approach in algae is that carbohydrates as a primary store of photosynthates can be exploited for biofuels through conversion to alcohols (Santelia and Zeeman, 2011). Algal polysaccharides can be hydrolyzed and then either fermented to ethanol by yeast or used as a heterotrophic carbon source for the production of a variety of biofuels (Harun et al., 2010). These carbohydrate productions in algae could be advantageous for biofuel production through overexpression of key enzymes in starch biosynthesis and secretion of soluble carbohydrates (Work et al., 2010).

### HYDROCARBONS

To overcome the challenges (e.g., limited supply and land yield, inconsistent performance, and challenging economics) for production of biodiesels derived from plant oils, microbial fatty acid esters production could be an alternative way (Reijnders, 2008; Work et al., 2010; Li et al., 2012). In contrast to chemical refining to obtain designed biodiesels, microbial fatty acid esters can be easily altered in their composition (Mayer and Shanklin, 2007) and degree of saturation through manipulation of key regulators of the fatty acid biosynthesis (Peralta-Yahya et al., 2012) and introduction of foreign genes encoding wax synthase/diacylglycerol acyltransferase from other microbes (Stoveken and Steinbuchel, 2008). In addition, when *Arabidopsis* fatty acyl-CoA reductases (Rowland et al., 2006), that catalyze the formation of a fatty alcohol from an acyl-CoA, were expressed in *E. coli, *more favorable short fatty alcohols were synthesized (Doan et al., 2009), indicating that more efficient fatty alcohol-producing enzymes might be paramount for the commercial production of fatty alcohols.

To improve the efficiency of electrical energy in storage, Li et al. (2012) reported a method to store electrical energy as chemical energy in higher alcohols, which used synthetic biology approach for converting electricity and CO_2_ to liquid biofuels. They genetically engineered a lithoautotrophic microorganism, *Ralstonia eutropha* H16, to produce isobutanol and 3-methyl-1-butanol in an electro-bioreactor using CO_2_ as the sole carbon source and electricity as the sole energy input. This recent breakthrough technology integrated with electrochemical formate production and biological CO_2_ fixation and higher alcohol synthesis, opens the possibility of electricity-driven bioconversion of CO_2_ to commercial chemicals.

As an ideal replacement for diesel fuel, fatty acid-derived hydrocarbons such as alkanes are produced directly from fatty acid metabolites in numerous organisms. Although two biochemical routes (decarbonylation vs. reduction of fatty alcohols) are available (Dennis and Kolattukudy, 1992; Wackett et al., 2007), their biosynthetic pathways to alkanes are not fully understood. Recently, Schirmer et al. (2010) performed comparative genomics and subtractive genome analysis with more than 50 genome sequences of cyanobacteria to find out two hypothetical enzymes and propose these as acyl-ACP reductase and aldehyde decarbonylase, responsible for alkane biosynthesis. Subsequently, their co-expression in *E. coli* enabled *in vivo* alkane biosynthesis (C13 to C17 mixtures), which is a major step toward the goal of low-cost renewable transportation fuels.

### NON-FERMENTATIVE SHORT-CHAIN ALCOHOLS

In contrast to fermentative alcohols produced from carbohydrates and lipids (**Figure 2**) like ethanol and butanol, the non-fermentative short-chain alcohols can be generated with protein sources by introducing exogenous transamination and deamination cycles (Huo et al., 2011). In order to develop a process for conversion of mixtures of peptides and amino acids to biofuels or chemicals, carbon skeletons could be provided from 2-keto acids through deamination of amino acids by 2-keto acid decarboxylases, and then to alcohols by alcohol dehydrogenases as shown in **Figure 2** (Atsumi et al., 2008). Alternatively, other amino acids could be deaminated to TCA cycle intermediates, which can be directed to pyruvate by malic enzymes or phosphoenolpyruvate carboxykinase. Pyruvate can be further extended to longer keto acids by acetohydroxy acid synthase or isopropylmalate synthase chain elongation pathways (Zhang et al., 2008). Such implementation of synthetic pathways together with rewiring metabolisms consequently enabled us to develop a protein-based process for biorefining and fuel production in potential platform cells such as *E. coli*, *B. subtilis*, yeast, and microalgae. Indeed, several *E. coli* variants that were improved for amino acid utilization were screened, and then isobutanol synthesis pathways were introduced into the cells to yield a strain that grew despite the stress generated by increased fuel production (Huo et al., 2011). Further, deletion of ammonium-assimilation genes, *gdhA *and *glnA*, increased the production of alcohols in the presence of the keto acid pathway. Thus, in contrast to previous metabolic engineering of carbon metabolic modules, application of nitrogen metabolic modules can be a significantly considerable alternative strategy in biofuel metabolic engineering.

### H_2_

Biological hydrogen (H_2_) is a potentially favorable, renewable, and ideal fuel for future demand in terms of climate change and sustainability. At present, biological H_2_ has been produced through three major processes: biophotolysis, photofermentation, and dark fermentation (Oh et al., 2011). In green algae and cyanobacteria, photosynthesis coupled to H_2_ evolution requires only water and sunlight (Prince and Kheshgi, 2005). Several cyanobacteria utilize an indirect pathway wherein storage carbohydrates generated through photosynthesis are fermentatively used to produce H_2_. Significant improvements for H_2_ production yield could be achieved by truncation of light-harvesting complexes and reduction of chlorophyll content in green algae and cyanobacteria (Beckmann et al., 2009; Mitra and Melis, 2010; Srirangan et al., 2011). Impaired cyclic electron transport also results in increased electron flow to the hydrogenase (Kruse et al., 2005a). Further findings of alternative anaerobic pathways (Hemschemeier and Happe, 2011), auxiliary electron transport (Peltier et al., 2010), and other distinct anaerobic H_2_ metabolisms (Meuser et al., 2009) could be potential targets to improve H_2_ production. Most hydrogenases are extremely oxygen-sensitive, which is one of the greatest challenges facing the establishment of an industrial biophotolysis process. For this reason, either control of O_2_ levels (Surzycki et al., 2007) or use of protein engineering should be implemented to create enzymes with greater O_2_ tolerance (Fritsch et al., 2011; Goris et al., 2011; Liebgott et al., 2011).

Alternatively, strictly anaerobic extremophiles, such as methanogenic archaea and hyperthermophilic bacteria, could be an attractive option for the production of biological H_2 _(Kim et al., 2010). H_2_ provides an excellent source of low potential reducing power for growth and biosynthesis of archaea (Sun et al., 2010). Recently, the heterologous expression in *E. coli* of a functional [NiFe]-hydrogenase from a hyperthermophilic archaea, *Pyrococcus furiosus*, was performed by employing a novel set of compatible vectors modified with an anaerobically induced *E. coli* hydrogenase promoter (Hopkins et al., 2011). Together with successful engineering of hydrogenase and nitrogenase (O_2_ sensitivity and higher molar production; Stripp et al., 2009; Yamamoto et al., 2009; Liebgott et al., 2010, 2011; Fritsch et al., 2011), development of versatile genetic systems and improvements in such oxygen-sensitive and intricate maturation requiring enzymes enable us to design and exploit the use of novel microorganisms and their constituent hydrogenases for biohydrogen production.

## PHOTOSYNTHESIS AND CO_2_ FIXATION

**FIGURE 3:**
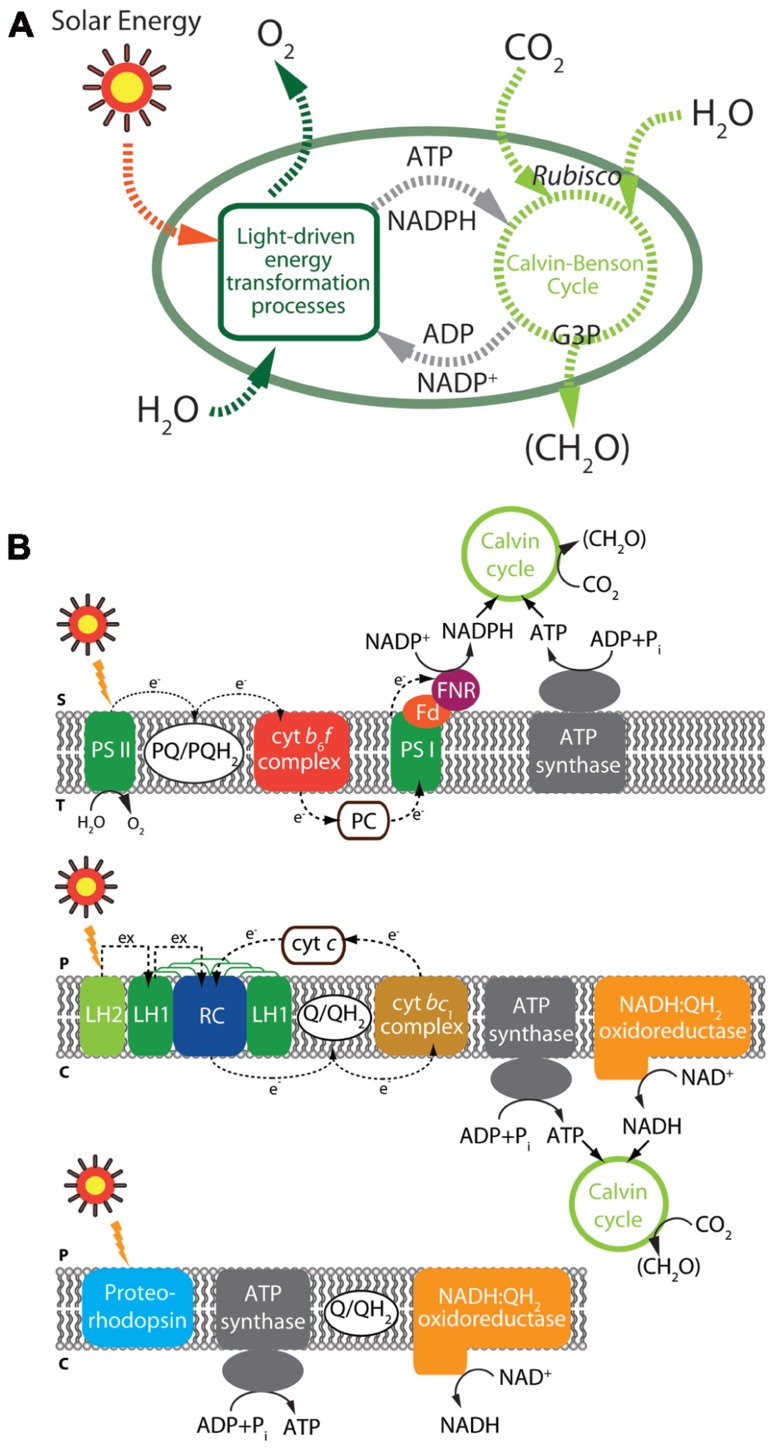
**Illustration of photosynthesis and carbon fixation **(A)** and key light-driven processes in microorganisms **(B)****. Black solid and dashed arrows represent reactions catalyzed by each enzyme and electron flows, respectively. (Top panel) Overview of oxygenic photosynthesis from cyanobacteria. (Middle panel) Overview of anoxygenic photosynthesis from α-proteobacteria. (Bottom panel) Light-driven proton pump. The major components of photosynthesis and carbon fixation including elements are depicted: S, chloroplast stroma; T, thylakoid lumen; P, periplasm; C, cytoplasm; Cyt *c*, cytochrome *c*; Cyt *bc*_1_, cytochrome *bc*_1_; Cyt *b*_6_*f*, cytochrome *b*_6_*f*; Fd, ferredoxin; FNR, ferredoxin-NADP^+^ reductase; LH, light-harvesting complex; PC, plastocyanin; PS, photosystem; PQ, plastoquinone; Q, ubiquinone; QH_2_, ubiquinol.

Solar energy-derived biofuels are one of the most abundant and favorable energy resources with respect to carbon neutralization and sustainability. The photosynthetic process, comprising the reduction of CO_2_, utilizing light and water by plants and algae (**Figure 3**), conserves solar energy in the form of reduced carbon compounds at a rate of approximately 120 TW, far exceeding the current annual global energy demand of approximately 14.9 TW (Barber, 2009; Hambourger et al., 2009). Hence, many researchers have focused on utilization of marine algae as a potential source of sustainable energy for biofuels, which can contribute to global energy independence (Robertson et al., 2011).

Photosynthetic microorganisms can serve as feedstocks for biofuels (Dismukes et al., 2008; Radakovits et al., 2010; Wijffels and Barbosa, 2010). Toward this aim, pathway engineering and culture modification have been developed with a result of high yields of biohydrogen, lipids, and carbohydrates. Nevertheless, the photosynthesis-derived biofuels with green algae still have significant challenges in the inefficiency of photosynthesis (Kruse et al., 2005b; Barber, 2009; Blankenship et al., 2011), the productivity of biomass, and the availability of genetic manipulation (Hambourger et al., 2009; Stephenson et al., 2011). In particular, optimization of light capture, energy transfer, and carbon fixation through manipulation of these pathways is essential for improvements in their photosynthetic efficiency, which is the principal determinant of productivity (Work et al., 2012). Several recent approaches by truncation or deregulation of light-harvesting antenna complexes (Beckmann et al., 2009; Melis, 2009), implementation of C4-type carbon concentrating mechanisms into C3 plants (Yamano et al., 2010; Reinfelder, 2011), optimization of the Calvin cycle by modulating expression levels of related enzymes (Miyagawa et al., 2001; Zhu et al., 2007), and expanding the solar spectrum (Chen et al., 2010; Chen and Blankenship, 2011) seem to be quite successful. For example, engineering and overexpression of ribulose-1,5-bisphosphate carboxylase oxygenase (RUBISCO) is a clear target for raising the efficiency of light energy conversion, resulting in productivity improvements (Lefebvre et al., 2005; Whitney et al., 2011).

In addition, lipids (i.e., triacylglycerols) in microalgae serve as an attractive biofuel feedstock, which can be converted to biodiesel through not only transesterification in biorefining (He et al., 2012) but also genetically interrupting starch biosynthesis (Wang et al., 2009b; Work et al., 2010). Accordingly, several attempts have been made to increase the lipid productivity of algae by screening and/or engineering algae using new enabling technologies, such as whole-genome sequencing, transcriptomics, metagenomics, and flow cytometry (Scott et al., 2010; Work et al., 2010; Stephenson et al., 2011). In addition, cyanobacteria as an excellent system for biodiesel production have been engineered by overexpression of a bacterial diacylglycerol acyltransferase, a phosphatidate phosphatase, and an acetyl-CoA carboxylase for increased lipid production (James et al., 2010). Other important and reasonable strategies for photosynthesis with microalgal biofuels (e.g., truncation of antenna complexes and lipid accumulation) are well described in (Wijffels and Barbosa, 2010; Work et al., 2012).

Alternatively, production of chemicals and fuels directly from CO_2_ is an attractive approach to solving energy and environmental problems (Lan and Liao, 2011). Previously, production of 1-butanol as a potential fuel substitute and an important chemical feedstock by the fermentative coenzyme A (CoA)-dependent pathway (Ezeji et al., 2007; Papoutsakis, 2008) using the reversal of β-oxidation has been performed in various organisms (Nielsen et al., 2009; Nicolaou et al., 2010; Yu et al., 2011). In addition, a modified clostridial 1-butanol pathway, including synthetic build-up of NADH and acetyl-CoA, enabled *E. coli* cells to produce a high titer and a high yield of 1-butanol production (Shen et al., 2011). Subsequently, the thermodynamically unfavorable step of the condensation of acetyl-CoA to acetoacetyl-CoA could be driven by artificially engineered ATP consumption through a pathway modification together with substitution of bifunctional aldehyde/alcohol dehydrogenase with separate butyraldehyde (butanal) dehydrogenase and NADPH-dependent alcohol dehydrogenase to improve the direct photosynthetic production of 1-butanol from cyanobacteria *Synechococcus elongatus* PCC 7942 (Lan and Liao, 2012). Therefore, these approaches, based on the importance of ATP and cofactor driving forces, have made a novel avenue to designing an efficient principle to alter metabolic flux by use of non-natural pathways.

## FUTURE PERSPECTIVE

A wealth of genome information dramatically expands our understanding of a variety of microbial metabolic pathways available for our purposes. This leads us to attempt to design and engineer microbial cell factories devoted to producing high yields of biofuels by treating metabolic pathways as modules or parts that can be readily moved at will from one organism to another. To date, many successful examples of implementation of non-indigenous metabolic or enzymatic modules in microbial host cells have been made through redesign and rearrangement of pathways, and the creative engineering of metabolic enzymes. Nonetheless, there are still limitations to obtaining the theoretical maximal yields of biofuels to meet our practical demands. Toward these aims, we need to further understand how microbial cells can coordinate their metabolic pathways under different environmental conditions, underlying essential and non-essential genes for bacterial life and metabolic networks. This can provide an effective direction for the design of minimal maintenance energy in cells. Moreover, balancing metabolic fluxes between biosynthesis of cellular mass and production of biofuels through modulation of their metabolic efficiency in cells can be an important key factor to achieving high yields of biofuel productions. Hence, both aspects are directly correlated to the choice of feedstock and biofuel-producing pathways, which is the fundamental basis for the cost-effective production of biofuels in high yield.

Overall, to design and construct the ideally synthetic microorganisms for biofuel productions, most desirable and effective are both (i) the increased efficiency of biosynthesis by the reduction of unnecessary energy-transducing components and their coordinated metabolic pathways, and (ii) high yields of biofuel production by the implementation of non-indigenous pathways with which renewable energy is ultimately transferred to the conversion of usable biomass and/or CO_2_ to biofuels.

## Conflict of Interest Statement

The authors declare that the research was conducted in the absence of any commercial or financial relationships that could be construed as a potential conflict of interest.
